# Predictors of Childhood Exposure to Parental Secondhand Smoke in the House and Family Car

**DOI:** 10.3390/ijerph6020433

**Published:** 2009-02-02

**Authors:** Vassiliki Mantziou, Constantine I. Vardavas, Eleni Kletsiou, Kostas N. Priftis

**Affiliations:** 1 National and Kapodistrian University of Athens, Department of Nursing, Athens, Greece; Emails: vmatziou@nurs.uoa.gr (V. M.); eklets@nurs.uoa.gr (E. K.); 2 Department of Social Medicine, Faculty of Medicine, University of Crete, Greece; 3 Department of Allergy and Pneumonology, Children’s Hospital of Pentelis, Athens, Greece; Emails: kpriftis@otenet.gr (K. P.)

**Keywords:** Passive smoking, environmental exposure, preschool children, smoking, determinants, parents, Greece, home exposure, behavior

## Abstract

Childhood exposure to secondhand smoke (SHS) is a serious threat to public health and can be influenced by parental lifestyle habits and beliefs. Taking the above into account we aimed at locating predictors of parental induced exposure to SHS in the house and family car among 614 children who visited the emergency department of two large pediatric hospitals in Athens, Greece. The multivariate analysis revealed that the factors found to mediate household exposure to paternal SHS were the number of cigarettes smoked per day (O.R 1.13, *p*<0.001) while, having a non-smoking spouse had a protective effect (O.R 0.44, *p*=0.026). Maternally induced household SHS exposure was related to cigarette consumption. For both parents, child exposure to SHS in the family car was related to higher numbers of cigarettes smoked (*p*<0.001), and for fathers was also more often found in larger families. Additionally, lower educated fathers were more likely to have a spouse that exposes their children to SHS inside the family car (O.R 1.38 95%C.I: 1.04–1.84, *p*=0.026). Conclusively, efforts must be made to educate parents on the effects of home and household car exposure to SHS, where smoke free legislation may be difficult to apply.

## 1. Introduction

Children’s exposure to second hand smoke (SHS) is a serious threat to public health and is a potent mixture of carcinogens, volatile toxins and chemicals [1,2]. It has been estimated that at least 1,000 million adults are smokers worldwide and that at least 700 million children breathe air polluted by tobacco smoke at home [3].

Exposure to cigarette smoke during childhood is related to the ever increasing frequency of childhood diseases such as respiratory illness, asthma, otitis media, and sudden infant death syndrome [4,5] and may lead to a predisposition towards vascular dysfunction and cancer [6,7] a fact that comes to stress the ever growing necessity for smoke-free households and cars.

Children’s exposure to SHS is usually involuntary, arising from smoking mainly by adults, in the places where children live and play, with an emphasis on SHS exposure in the home or the household car. [8,9] In places and households where people smoke, infants are not only directly exposed to SHS toxins through its direct inhalation, but also through other pathways of exposure including the inhalation and ingestion of contaminated dust and skin contact with both smokers and contaminated household surfaces [10]

The investigation into the specific parental characteristics that are related to childhood SHS exposure is of interest, as it seems that these characteristics differ between different social groups and between different nationalities with the majority of findings focused towards maternal smoking habits [11,12]. Previous research has indicated that predictors of SHS exposure during childhood are factors such as the parent’s social class and educational level, the season of the year (winter has higher levels of SHS exposure), the household dimensions, the day of sample collection, the number of children in the family as also the child’s gender, while medically at risk children are among the most venerable populations towards SHS exposure [9–17].

Taking the above into account, the purpose of this study was to investigate into the possible predictors of childhood SHS exposure due to parental smoking, and to investigate into the relationships between the parents’ characteristics and their children’s exposure to cigarette smoke inside the house or family car among a pediatric population in Greece.

## 2. Methodology

### 2.1. Study Design-Study Population

The study population was comprised of 662 children, younger than 12 years of age (342 males and 320 females, with a mean age 4.8 ± 2.5 years) that visited the Emergency Departments of the two Pediatric Hospitals of Athens “Aghia Sophia” and “Aglaia Kyriakou” between September and December, 2004. These two pediatric hospitals cover almost the entire child population within the area of Attica, (which includes the city of Athens), while many children from other parts of Greece are often hospitalised in these institutions as the Greek health care system allows patients to obtain health care from any public hospital of their choice.

Data was collected from the children’s mothers through personal interviews, with the use of a structured questionnaire that investigated into parental demographic, social and educational characteristics in relationship to smoking in the house or family car.

Ethical approval was obtained from the Bioethics board of the “Agia Sofia” hospital (192525/02), while written consent was obtained from the mothers prior to completing the questionnaire. No mother refused to participate (response rate of 100%) however; some mothers did not answer some of the questions. Exposure to household SHS was defined as “as least one of the parents smokes in front of their child ≥1 cigarette per day”.

### 2.2. Sample Size Estimation

The adequate number of people for investigating into the relationship between the independent variables with the frequency of exposure to SHS was estimated. For each test with a p-value of 0.05 and statistical power (1-β) of 0.80, and according to our estimations at least 47 people were needed. Subsequently, for testing the 13 associations included in the questionnaire, at least 611 people were estimated to be needed. The week that the limit of 611 persons was exceeded, collection of data stopped therefore finalizing the total number of participants at 662.

### 2.3. Statistical Analysis

The statistical analysis was performed with the use of the statistical package SPSS 16.0 (SPSS Inc., Chicago, IL). In order to explore the relationship between parental characteristics and child exposure to cigarette smoke, two-sided tests were performed. Continuous variables are presented as mean ± standard deviation, while qualitative variables are depicted with the use of frequencies. Initially, a univariate analysis was performed so as to investigate into whether one or more of the parents’ and children’s characteristics were related to the existence of parental induced SHS exposure. In addition to the above univariate model a backward stepwise logistic regression model was applied so as to investigate into the main determinants of childhood exposure to parental SHS in the house or family car. Odds Ratio (OR) and 95% confidence intervals (95%CI) were also derived. Relationships with a p-value (*p*) ≤0.05 were considered as statistically significant. The variables entered during step 1 of the stepwise backward logistic regression analysis model include: the child’s gender, child’s age, number of children in the family, the father’s educational status (low<12 years education vs. high >12 years education), the mother’s educational status (low<12 years education vs. high >12 years education), the father’s and mother’s number of cigarettes per day, whether friends or relatives are allowed to smoke in the house, the belief that they do not want their child to become a smoker and the spouse’s smoking status (modified in each case according to whether maternal or paternal characteristics were investigated into).

## 3. Results

### 3.1. Descriptive and Demographic Characteristics

The study population’s descriptive characteristics are depicted in Table 1 below. Children of pre-school age (4–6 years old) comprised the largest age group (47%), followed by toddlers of 1–3 years (31.7%). Sixty six percent of the fathers and 50% of the mothers were smokers with an average consumption of 16.3 and 9.3 cigarettes per day. The parental smoking habits were similar in regards to only the type of tobacco consumed as almost all participants consumed cigarettes (except two men, who smoked cigars), while when investigating into the percentage of households that were exposed to parental smoke (mother or father a smoker) 69.2% of households had at least one parent that smokes in front of their child, exposing it to SHS, while in 19.6% of the households both parents’ reported smoking in front of their children.

In addition to the above, the mean age was 4.8 years for both males and females (*p*=0.813). Fathers on average were older (36.4 vs. 32.1 years) and had a higher level of education than mothers with 71.9% percent of the fathers having a higher university education, in comparison to 58.2% of the mothers.

### 3.2. Factors Related to Parental Induced SHS Exposure in the Car and House

To investigate into the parental characteristics which were associated with reports of household SHS exposure (None vs. reported household exposure to parental SHS), initially a univariate analysis was performed. Specifically the fathers’ level of education, parental smoking habits, the mothers’ age and the number of smokers in the house were found to be significantly related (*p*<0.05) to household exposure to parental SHS as seen in Table 2.

In addition to the univariate analysis used to investigate into the predictors of household SHS exposure a second univariate analysis was performed to investigate into the characteristics that were related with childhood exposure to SHS in the family car (data not shown). The analysis revealed that the higher the number of cigarettes smoked per day by the father (*p*<0.001) and the larger number of children in the family were significantly associated to exposure to paternal related smoking in the car (*p*=0.019). On the contrary the fathers level of education (*p*=0.507), the child’s gender (p=0.924), the child’s age (*p*=0.687), and the fathers age (*p*=0.714), were not found to be significantly associated with paternal smoking in the car. Similar findings were also found when investigating into the relationship between maternal characteristics and maternal smoking in the family car, with only the number of cigarettes smoked per day found to be associated with parental SHS in the family car, while the mothers age (*p*=0.227) and level of education (*p*=0.575) as also the child’s gender (*p*=0.469) and age (*p*=0.471), were not related to maternal self reported smoking in the car.

### 3.3. Paternal Characteristics Found to Mediate Childhood SHS Exposure in the House and Family Car

The multivariate logistic regression analysis provided a clearer insight into the actual characteristics that influence paternal induced childhood exposure to SHS in the house and family car. According to the performed backward stepwise multivariate analysis, (Table 3), household SHS exposure due to paternal smoking was associated with the number of cigarettes smoked per day the father (*p*<0.001), the child’s age (younger children were more likely to be exposed to paternal smoke) (*p*=0.026) and if his spouse is a non smoker (O.R 0.44 95%C.I: 0.24–0.80, *p*=0.007), additionally a trend was noticed for lower educated fathers to be less likely to expose their children to SHS in the house in comparison to their higher educated peers (O.R 0.57 95%C.I: 0.30–1.07, *p*=0.077).

An additional regression analysis was performed so as to reveal the characteristics that would influence paternal smoking in the family car with children as passengers. Again the number of cigarettes consumed per day by the father affected childhood SHS exposure (*p*<0.001), as also the number of children in the family (B coefficient = 0.33, *p*=0.026). Although a trend was noticed for the spouses lower educational status to have a protective effect on paternal smoking habits in the car (controlling for her smoking status), this relationship did not reach the point of statistical significance.

### 3.4. Maternal Characteristics Found to Mediate Childhood SHS Exposure in the House and Family Car

The characteristics that wee found to influence childhood exposure to SHS due to the mothers smoking habits as seen in Table 4. The only factor that was found to significantly influence maternal smoking inside the house or family car in the presence of her children were the number of cigarettes smoked by the mother per day (*p*=0.019 and *p*<0.001 respectively), while a tendency for lower educated mothers to expose their children higher to SHS was also noted although it did not reach the level of statistical significance (O.R 1.51 95%C.I: 0.95–2.40, *p*=0.083). While the fathers educational status was not associated with maternal smoking habits in the house, a borderline tendency for his educational status to influence maternal smoking in the car was noticed (*p*=0.054) with mothers who smoke inside the car more likely to have a lower educated spouse (O.R 2.23, 95%C.I: 0.99–5.01, *p*=0.054).

## 4. Discussion

### 4.1. Main Findings

According to our study’s findings, a high percentage of Greek households and cars were not found to be smoke-free. The noted predictors of household SHS exposure due to maternal or paternal smoking were the number of cigarettes smoked by both the mother and father per day and the child’s age, while the mothers’ educational level was borderline not statistically related to SHS household exposure. As for parentally induced SHS in the family car, again the number of cigarettes smoked per day was found to be a strong predictor of SHS exposure as also the number of children in the family. In addition to the above, the other parent’s educational status seemed to affect the spouses habits in the family car with lower educated husbands found to be more likely to have a spouse that smokes in the family car with their children present.

The percentage of parents that smoke in our study were substantially higher than both the prevalence of smoking at a national level as also the percentage of parents of preschool children that smoke [9,18]. A large population based study spanning the prefecture of Chania, Crete indicated that 63% of households with children of our age group, had at least one parent a smoker [8] (52% of the fathers and 36% of the mothers were active smokers), lower than the 69.2% within our hospital based study population, a factor indicative of the effect household exposure to SHS has on ones health [8]. It is also alarming that in almost 20% of the households both parents reported smoking in front of their children, while almost all of the fathers who smoked were reported to expose their children to SHS.

### 4.2. Household SHS Exposure

We identified the fact that both maternal and paternal smoking habits have a strong effect on child SHS exposure, with the number of cigarettes smoked by the parent directly associated with exposing their children to SHS. Using quantitative calculations and biomarker assessment, childhood SHS exposure has been positively associated with the number of cigarettes smoked by both parents, separately and in total, with maternal smoking habits found to play a more important role in exposure during early childhood [13]. Paternal smoking in the household was influenced by the child’s age, with younger children less likely to be exposed to SHS, a finding similar to findings from other studies, which indicate that the age of the youngest child in the household is a strong predictor of the presence and uptake of a smoke-free home [19,20]. Although research has indicated that a low educational status has also been related to the regression of smoking bans in the household [19], it is a paradox that we found higher educated fathers to be more likely to smoke in front of their children in comparison to their less educated peers. This regional difference could be attributed to disparities in socioeconomical status, a fact that we could not take into account during the analysis. Although it has been found in Greece that higher educated mothers and those of higher socioeconomical status are more likely to be smokers, to our knowledge this has not been found for Greek males yet [17].

Pizacani *et al.*, had indicated that full household smoking bans, and thus lower SHS exposure, were strongly associated with awareness of the harm of SHS [21]. We found that among mothers that smoke and controlling for their spouses smoking habits, a tendency for less educated mothers were one and a half times more likely to smoke in front of their children in comparison to their higher educated peers, a fact that could be indicative of the lack of knowledge that Greek mothers have on the effect SHS has on their child’s health and development.

Parental smoking in the home, not only influences childhood SHS exposure but can also lead to the social acceptance of smoking by the child. It is documented that parents play an important role as role models and can influence substance use and health beliefs, as parental smoking status has been recognised as a strong predictor of smoking experimentation during childhood [22], Additionally, exposure to their parents’ smoking habits may lead adolescents to develop the perception that smoking is a normative adult behaviour [23], and in correlation with social norms and peer pressure may additionally promote tobacco use and experimentation among youth.

### 4.3. Family Car SHS Exposure

Air monitoring studies have documented the extremely high levels of SHS exposure one is exposed to, when a passenger in a car smokes [24,25]. Children and infants, due to their higher ventilation rates and smaller body mass, inhale elevated levels of SHS for the same level of exposure, and thus are more vulnerable during the short but intense exposure to SHS, within the family car. Due to the above fact, there are strong ethical and public health arguments for governmental interventions and regulations, despite the ongoing debate on the ethics of making private vehicles mandatory smoke-free [26]. Research has indicated the strength and necessity of implementing smoking bans in cars as the number of cigarettes smoked in the car and the number of cigarettes smoked by the owner are associated with nicotine contamination of the interior, while air nicotine levels in smoker cars with bans were not significantly different from those found in nonsmoker cars [27]. Previous research among German preschool children’s exposure to SHS in their family car has indicated that the family’s large size and higher parental education were factors that reduced childhood SHS exposure [28]. Our findings are not identical but similar as although we too found mothers twice more likely to smoke in the family car if their spouse was of a lower level of education in comparison to women with higher educated husbands. Despite the above noticed trend (without it reaching the level of statistical significance), we on the contrary found that husbands were less likely to smoke in the family car if their spouse was of a lower educational level, a fact that we could not explain. Further research is warranted to investigate into the above paradox.

### 4.4. Main Strengths and Limitations

We must state that the ideal method of evaluating SHS exposure, using the biomarker cotinine, the main metabolite of nicotine, was not performed during this study and the results are based on self reported household exposure to SHS and thus subject to recall and response bias. Biomarker assessment would have permitted a more in depth quantitative analysis into the factors that predict household SHS exposure, opposed to the current qualitative analysis. The conclusions drawn in this manuscript are based on cross sectional survey data, and are thus limited in ability to attribute cause and effect. During the analysis and due to insubstantial data we were not able to investigate neither into inter-hospital clustering nor into the ages of the participants’ siblings, two factors that we must note as a limitation and should be taken into account when designing studies that investigate into SHS exposure.

Despite the above, the use of the two largest pediatric hospitals in Athens, and the statistically derived sample size allow us to interpret with confidence the results and compare them to those of other qualitative SHS exposure studies. Further research is needed with the use of biomarker assessment so as to investigate into any possible “dose-response” effects and would allow for a more in-depth analysis of the determinants that modify household and family car SHS exposure among children in Greece.

## 5. Conclusions

Parental smoking habits and the number of children in the family were found to be predictors of parentally induced SHS exposure among Greek children in both the household and family car. As a large percentage of public places in Greece will go smoke free within 2009, efforts must be made to inform and educate Greek families on the effects of household exposure to SHS, where smoke free legislation is difficult to apply. The identification of these parental characteristics provides valuable information for the configuration of both mass media and community based educational and prevention programs, which when applied in coherence with nation wide smoke free legislation, may lead to a noted reduction in childhood SHS exposure [29,30].

## Figures and Tables

**Table 1.** Descriptive characteristics of the study population.

Variable	Data
Children’s’ gender *% (n)*	
*Boy*	51.7 (342)
*Girl*	48.3 (320)
Children’s’ age category *% (n)*	
*0–3*	31.7 (210)
*4–6*	47.1 (312)
*7+*	21.1 (140)
Smokers % (n)*	
*Father*	66.3 (439)
*Mother*	50.0 (331)
Number of cigarettes smoked/day Mean ± SD*	
*Father*	16.3 ±12.8
*Mother*	9.32 ± 9.9
Parent smokes inside the house around children % (n/N) *	
*Father*	85.4 (375/439)
*Mother*	57.1 (189/331)
Parentally induced SHS exposure in the household *% (n)*	
*No parents*	30.8 (204)
*One parent*	49.5 (328)
*Both parents*	19.6 (130)
Parent smokes inside the car when children are inside % (n/N) *	
*Father*	56.5 (248/439)
*Mother*	10.3 (34/331)

n/N: positive answers/total answers, SD= standard deviation;

* Smoking participants only

**Table 2.** Characteristics associated to the self reported SHS exposure in the home in the univariate analysis.

Variable	Non-exposed	SHS-exposed	p-value
Childs age*mean±SD	5.0 ±2.9	4.7 ± 2.3	0.066
Fathers age*	37.2±6.5	36.1±6.7	0.050
Mothers age*	32.9±5.8	31.7±5.9	**0.023**
Number of smokers in the house*	0.6±0.6	1.5±0.5	**<0.001**
Number of children*	1.8±0.7	1.9±0.7	0.314
Paternal cigarettes per day*	6.1±10.2	20.9±11.2	**<0.001**
Maternal cigarettes per day*	4.3±7.6	11.6±10.0	**<0.001**
Childs gender**			
*Male*	34.2 (117)	65.8 (225)	**0.05**
*Female*	27.2 (87)	72.8 (233)	
Housing**			
*Apartment building*	29.3 (127)	70.7 (307)	0.372
*Free standing house*	32.9 (74)	67.1 (151)	
Maternal education**			
*Lower*	31.8 (87)	68.2 (187)	0.733
*Higher*	30.4 (116)	69.6 (265)	
Paternal education**			
*Lower*	41.6 (77)	58.4 (108)	**<0.001**
*Higher*	26.4 (125)	73.6 (349)	
Friends/relatives smoke at home**			
*Yes*	28.8 (115)	71.2 (284)	0.164
*No*	34.1 (86)	65.9 (166)	
Bothers you if your child becomes a smoker**			
*Yes*	32.5 (160)	67.5 (333)	0.124
*No*	26.0 (44)	74.0 (125)	

* Student t-test, equal variances assumed, results depicted as mean±standard deviation

** Chi-squared test, exact, two sided, presented as % (n)

**Table 3.** Adjusted Odds Ratio of the paternal characteristics that were found to be significantly associated with childhood exposure to SHS due to parental smoking*.

	B coefficient	O.R	95% C.I.		p-value
			Lower	Upper	
Paternal smoking in the house in front of the children**					
Paternal Educational status (Low)	−0.57	0.57	0.30	1.07	0.077
Paternal cigarettes per day	0.12	1.13	1.08	1.19	***<0.001***
Spouse smokes (No)	−0.82	0.44	0.24	0.80	***0.007***
Childs age	−0.12	0.89	0.80	0.99	***0.026***
Paternal smoking in the family car with children as passengers**					
Maternal Educational status (Low)	−0.36	0.78	0.46	0.69	0.078
Paternal cigarettes per day	0.06	1.06	1.03	1.09	***<0.001***
Number of children in the family	0.33	1.38	1.04	1.84	***0.026***

*. Smoking fathers only (n=439)

**. A Stepwise backward logistic regression model was applied. Variables entered on step 1: child’s gender, child’s age, number of children, father’s educational status, mother’s educational status, father’s number of cigarettes per day, spouse smoker (no vs. yes), friends or relatives allowed to smoke in the house, belief that they do not want their child to become a smoker.

**Table 4.** Adjusted Odds Ratio of the maternal characteristics that were found to be significantly associated with childhood exposure to SHS due to maternal smoking*.

	B coefficient	O.R	95% C.I.		p-value
			Lower	Upper	
Maternal smoking in the house in front of the children**					
Maternal Educational status (Low)	0.41	1.51	0.95	2.40	0.083
Maternal cigarettes per day	0.04	1.05	1.00	1.08	***0.019***
Maternal smoking in the family car with children as passengers**					
Paternal Educational status (Low)	0.80	2.23	0.99	5.01	0.054
Maternal cigarettes per day	0.16	1.17	1.09	1.26	***<0.001***

*. Smoking mothers only (n=331)

A Stepwise backward logistic regression model was applied. Variables entered on step 1: child’s gender, child’s age, number of children, father’s educational status, mother’s educational status, father’s number of cigarettes per day, spouse smoker, friends or relatives allowed to smoke in the house, belief that they do not want their child to become a smoker.
